# Supplementary material for: Diet-Induced Obesity and NASH Impair Disease Recovery in SARS-CoV-2-Infected Golden Hamsters
Source: Viruses. 2022 Sep 17;14(9):2067. doi: 10.3390/v14092067 (PMC9503118; doi:10.3390/v14092067)
Supplement: Supplementary file 1 [file viruses-14-02067-s001.zip › viruses-1878575-supplementary.pdf]

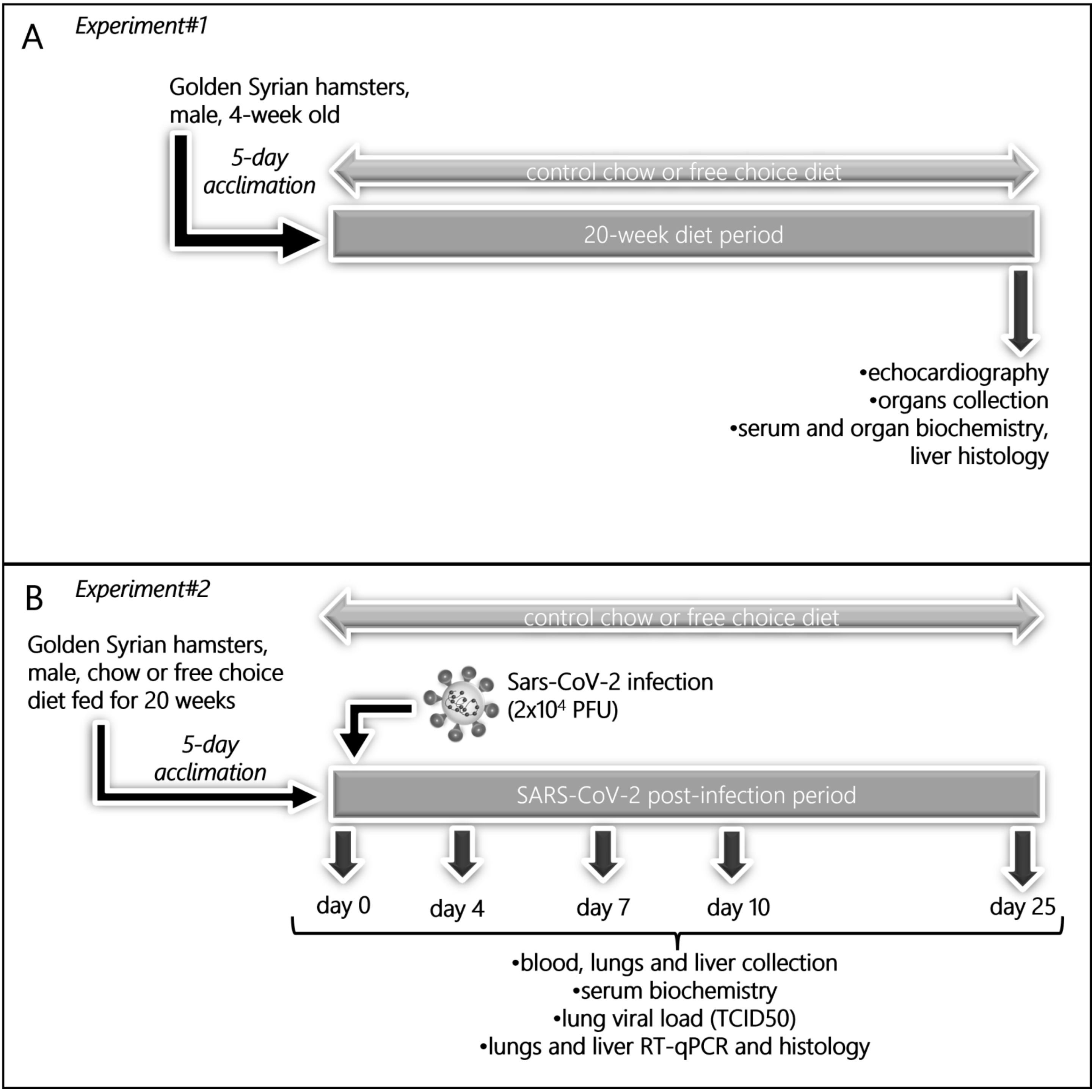

Figure S1: (A) Experiment #1 and (B) Experiment #2.

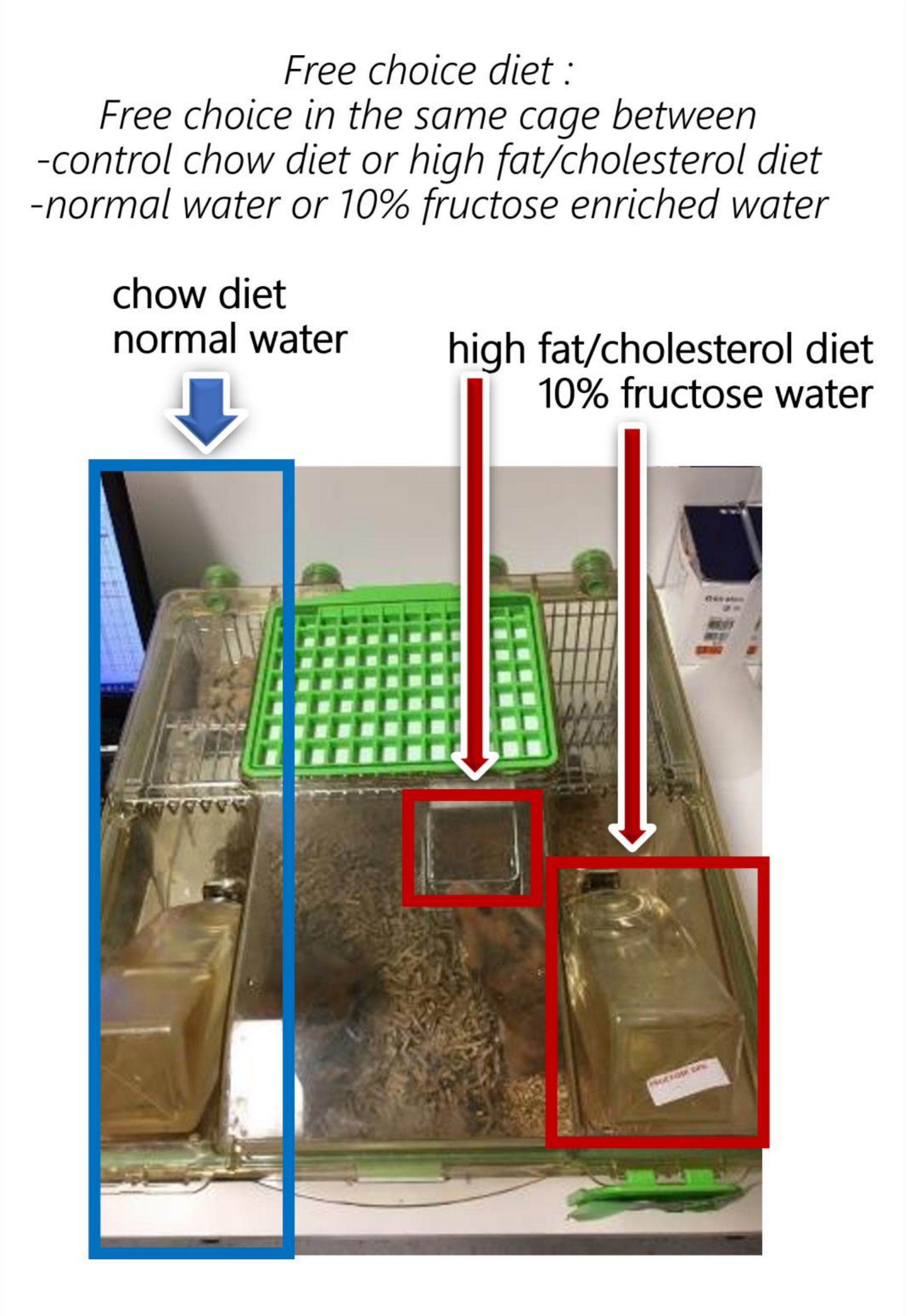

Figure S2: Cage setting for hamsters given a free-choice diet.

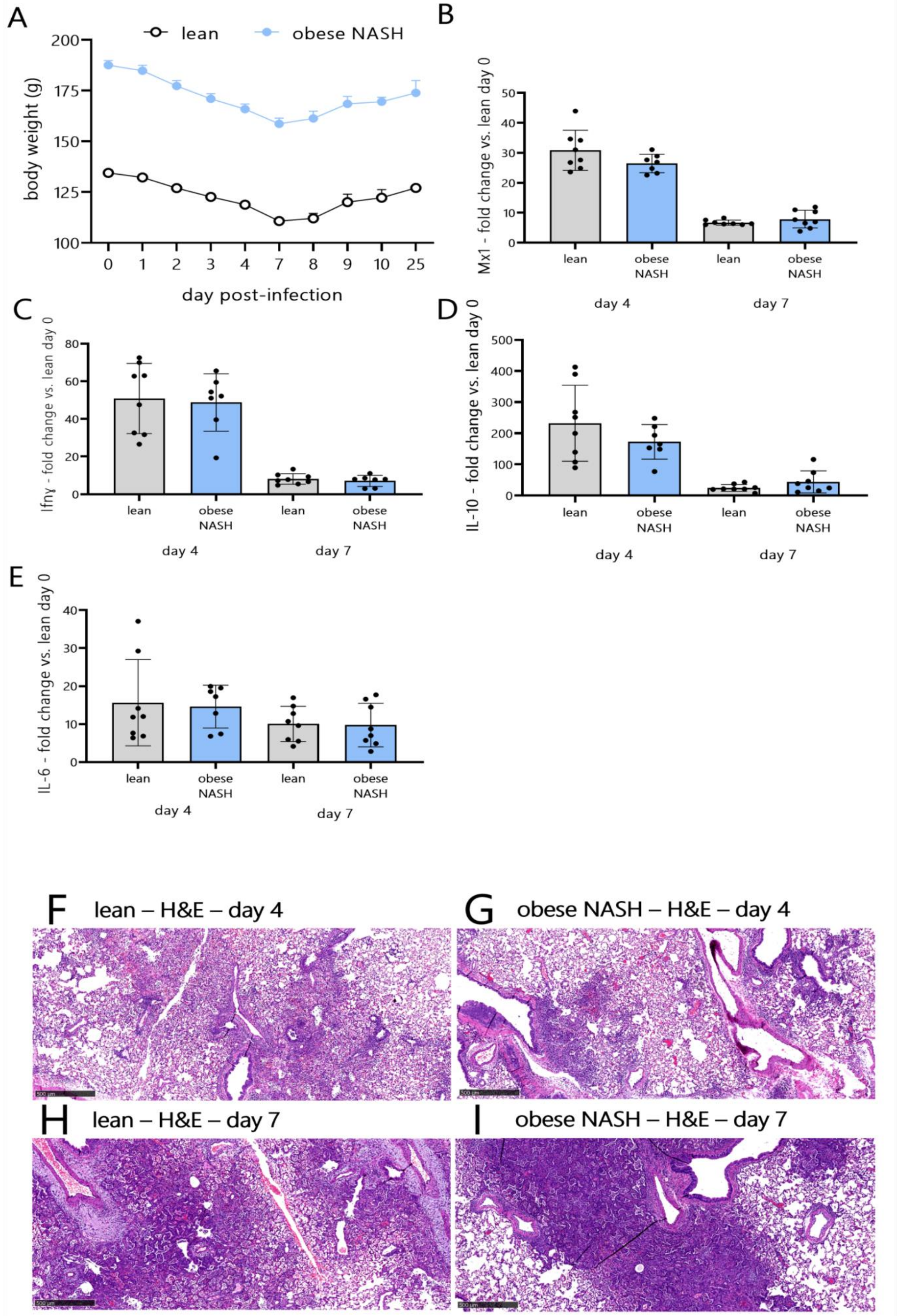

Figure S3. Lung gene expression and histologic features on dpi 4 and 7. (A) Changes in body weight (in g) are depicted. (B–E) The lungs of mock-infected and SARS-CoV-2-infected lean hamsters and obese NASH hamsters were collected on dpi 4 and 7. mRNA copy numbers were quantified by qRT-PCR. The data are expressed as the fold increase over the expression level in mock-treated animals (*n* = 6-8 animals/group). It should be noted that there were no significant differences between mock-treated lean hamsters and mock-treated obese NASH hamsters (day 0) (not shown). (F-I). Representative lung histology images (H&E staining). Scale bars: 500 µm

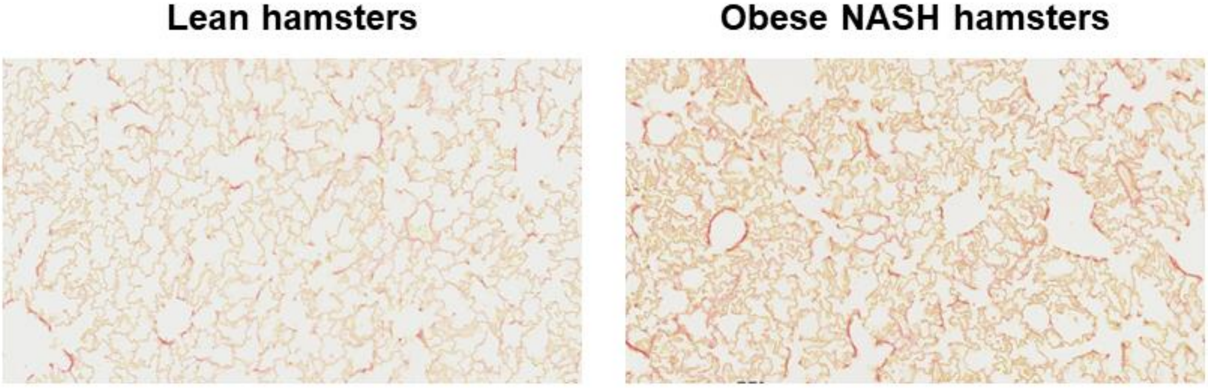

Figure S4. Histopathologic assessment of lung samples from lean hamsters and obese NASH hamsters at baseline. Representative images of sections stained with Sirius Red are shown.

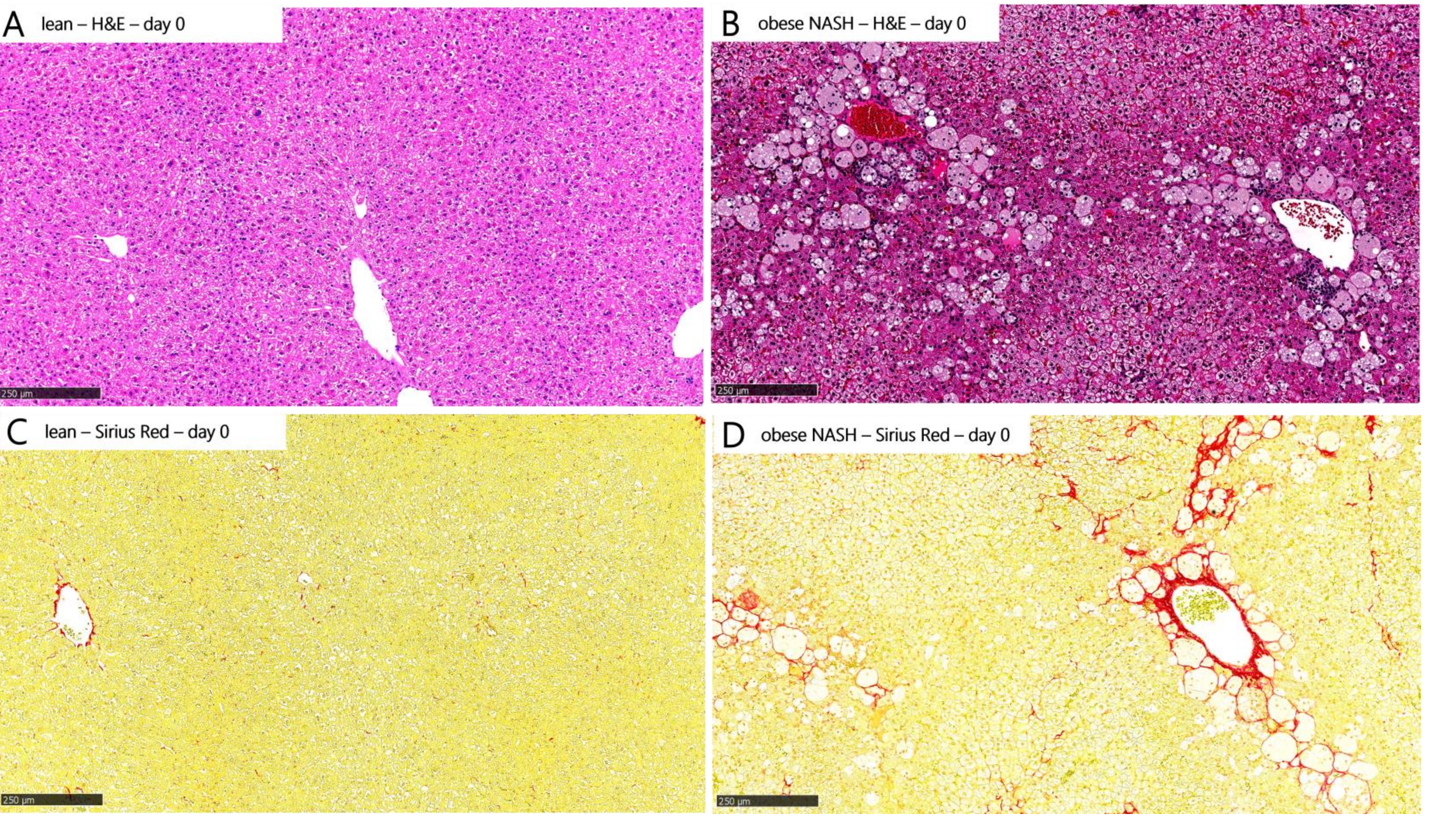

Figure S5. Histopathologic assessment of liver sections from lean hamsters (A,C) and obese NASH hamsters (B,D) at baseline. Representative images of sections stained with H&E and Sirius Red are shown.
